# The role of S100A4 for bone metastasis in prostate cancer cells

**DOI:** 10.1186/s12885-021-07850-4

**Published:** 2021-02-06

**Authors:** Bongjun Kim, Suhan Jung, Haemin Kim, Jun-Oh Kwon, Min-Kyoung Song, Min Kyung Kim, Hyung Joon Kim, Hong-Hee Kim

**Affiliations:** 1 Department of Cell and Developmental Biology, BK21 PLUS Program and DRI, School of Dentistry, Seoul National University, 101, Daehak-ro, Jongno-gu, Seoul, Republic of Korea 03080; 2 Current address: Department of Experimental Radiation Oncology, The University of Texas MD Anderson Cancer Center, Houston, TX 77030 USA; 3 Arthritis and Tissue Degeneration Program, David Z. Rosensweig Genomics Research Center, Hospital for Special Surgery, New York City, NY USA; 4 Department of Oral Physiology, BK21 PLUS Project, and Dental and Life Science Institute, School of Dentistry, Pusan National University, Mulgeum-eup, Yangsan, Busan, 50612 South Korea

**Keywords:** Prostate cancer, Bone metastasis, S100A4, Epithelial-mesenchymal transition, Osteoclast

## Abstract

**Background:**

Prostate cancers frequently metastasize to bone, where the best microenvironment for distant colonization is provided. Since osteotropic metastasis of prostate cancer is a critical determinant of patients’ survival, searches for preventive measures are ongoing in the field. Therefore, it is important to dissect the mechanisms of each step of bone metastasis, including the epithelial-mesenchymal transition (EMT) and cross-talk between metastatic niches and cancer cells.

**Methods:**

In this study, we established a highly bone-metastatic subline of human prostate cancer cells by selecting bone-homing population of PC3 cells after cardiac injection of eight-week-old male BALB/c-nude mice. Then we assessed the proliferation, EMT characteristics, and migration properties of the subline (mtPC3) cells in comparison with the parental PC3 cells. To investigate the role of S100A4, we performed gene knock-down by lentiviral transduction, or treated cells with recombinant S100A4 protein or a S100A4-neutralizing antibody. The effect of cancer cells on osteoclastogenesis was evaluated after treatment of pre-osteoclasts with conditioned medium (CM) from cancer cells.

**Results:**

The mtPC3 cells secreted a markedly high level of S100A4 protein and showed elevated cell proliferation and mesenchymal properties. The increased proliferation and EMT traits of mtPC3 cells was inhibited by S100A4 knock-down, but was not affected by exogenous S100A4. Furthermore, S100A4 released from mtPC3 cells stimulated osteoclast development via the cell surface receptor RAGE. Down-regulation or neutralization of S100A4 in the CM of mtPC3 cells attenuated cancer-induced osteoclastogenesis.

**Conclusion:**

Altogether, our results suggest that intracellular S100A4 promotes cell proliferation and EMT characteristics in tumor cells, and that secreted S100A4 activates osteoclastogenesis, contributing to osteolytic bone metastasis. Thus, S100A4 upregulation in cancer cells highly metastatic to bone might be a key element in regulating bone metastasis.

**Supplementary Information:**

The online version contains supplementary material available at 10.1186/s12885-021-07850-4.

## Background

An aging population with increasing prevalence of prostate cancer (PC) has become a global health care challenge [1]. PC predom [2] inantly metastasizes to bone, and treatment of bone metastasis has been a major concern over several decades [1]. Typically, bone metastasis of PC forms osteoblastic lesions; however evidence suggests that osteolytic components also contribute to tumor growth in bone [2, 3]. The osteolytic nature of PC bone metastasis arises from cross-talk between tumor cells and normal bone cells. Metastatic cells in the bone microenvironment support osteoclast activation directly or indirectly by inducing factors such as RANKL, the most potent osteoclastogenic factor, in osteoblasts [4]. In support of this notion, tumor-secreted molecules such as parathyroid hormone-related peptide (PTHrP) and interleukin 8 (IL-8) enhance osteoclast-mediated bone resorption, thereby increasing the local availability of bone matrix storage proteins such as transforming growth factor-β (TGF-β) [5]. Subsequently, the increasing concentration of TGF-β perpetuates tumor growth in the bone, leading to a vicious cycle between tumor and bone [4]. This emphasizes the role of osteoclasts in releasing tumor growth factors from the bone matrix; in addition, recent studies have shown that dormant tumor cells in the bone microenvironment are re-activated by osteoclasts [6, 7]. Based on these findings, unveiling cross-talk between tumor cells and osteoclasts is a valid approach to find a cure for bone metastasis.

The metastasis of cancer involves epithelial-mesenchymal transition (EMT), one of the crucial steps in cancer metastasis, at the primary site [8]. During EMT progression, the transcription factor SNAI1 directly represses E-cadherin, resulting in the loss of epithelial properties [9], while the expression levels of proteins with mesenchymal features, such as N-cadherin and vimentin, are up-regulated [10]. PC metastasis engages EMT for successful colonization of distant organs including bone [2, 11, 12]. Several lines of evidence have revealed that epithelial-mesenchymal plasticity plays a pivotal role in both PC metastasis and drug-resistance [13, 14]. Therefore, our understanding of EMT mechanisms should be deepened to overcome bone metastasis in PC.

S100 calcium-binding protein A4 (S100A4) is a member of the S100 calcium-binding protein family, which plays a crucial role in the progression of cancer metastasis [15, 16]. S100A4 has been reported to mediate proliferation, migration, invasion, and apoptosis in many types of tumor cells, including prostate cancer cells [16, 17]. Because of its presence in both intracellular and extracellular regions of tumor cells, its molecular function has been studied in various experimental settings. Intracellular S100A4 interacts with liprin, ezrin, and non-muscle myosin II to promote cytoskeleton rearrangement, which mediates cell migration and invasiveness [18–20]. In addition, S100A4 binds with p53, a tumor suppressor, interrupting its DNA binding activity [21]. The extracellular function of S100A4 also supports tumor progression. Extracellular S100A4 has been shown to induce angiogenesis, neurite outgrowth, and migration of astrocytic tumor cells [22–24]. In particular, S100A4 interacts with receptor for advanced glycation end products (RAGE) on the surface of cells to increase the invasive potential of melanoma, thyroid cancer cells, and colorectal cancer cells [25–27]. Recently, we have reported that S100A4 promotes osteolytic bone metastasis in breast cancer by regulating cell migration and osteoclastogenesis [28]. However, the molecular mechanisms of S100A4 in the progression of PC bone metastasis remains unclear. Moreover, the role of S100A4 in cross-talk between prostate cancer cells and bone cells has not been investigated to date.

In this study, we obtained a highly bone-metastatic subline of human prostate cancer cells using an in vivo selection method. Elevated S100A4 in this bone-metastatic subline promoted proliferation and EMT traits by intracellular functions, and cell migration to some extent through extracellular mechanisms. Moreover, S100A4 release from this bone-metastatic PC stimulated osteoclastogenesis through the cell surface receptor RAGE.

## Methods

### Animals

Animal experiments were approved by the Institutional Animal Care and Use Committee (IACUC) of Seoul National University. All the animals were kept in an SPF facility with consistent temperature (22 °C) and humidity (55%), and a 12-h light/dark cycle. The facility was operated by experienced zookeepers who were responsible for serving bedding material, food, and sterilized water, under the supervision of a veterinarian. 4 to 5 mice were housed in each cage. All the animals had free access to food and water. All mice were sacrificed by carbon dioxide asphyxiation as approved by the IACUC of Seoul National University. Five-week-old female ICR mice and eight-week-old male BALB/c-nude mice were purchased from OrientBio (Sungnam, Korea). ICR (*n* = 5) mice were used for bone marrow cell preparation. Bone marrow-derived macrophages were prepared as previously described [29]. Bone-metastatic cancer selection was described in our earlier publication [28]. The bone-metastasis rate of cancer cells by intra cardiac injection was approximately 20% in our previous study. Thus we used five mice for bone-metastatic cancer selection. Briefly, left ventricular cardiac injection was performed with 1 × 10^5^ PC3 cells injected into nine-week-old male BALB/c-nude mice (*n* = 5). After 8 weeks, metastasized cells in femurs and tibiae were flushed out from the bone marrow and expanded in culture dishes for 8 weeks. Then, the immortal cells were re-injected into the left ventricle for another round of in vivo selection (*n* = 5). We named the cancer cells harvested after the second round the mtPC3 cells. Each purpose of experiment was to obtain primary cells or bone-metastasized cancer cell. No control group was used.

### Reagents

Recombinant M-CSF and RANKL were purchased from PeproTech. Recombinant mouse S100A4 was purchased from Prospec. Recombinant OPG was purchased from R&D Systems. Antibodies against vimentin and c-Fos were purchased from Santa Cruz. Antibodies against N-cadherin and snail2 were obtained from Cell Signaling Technology. Antibodies for RAGE (DD/A11) were from Millipore. Anti-S100A4 antibody was purchased from Abcam. Anti-β-catenin antibody was purchased from Invitrogen. Antibodies for NFATc1 (7A6), E-cadherin, and hHLA were purchased from BD Pharmingen. Anti-β-actin antibody (AC-74) and the leukocyte acid phosphatase kit (for TRAP staining) were purchased from Sigma-Aldrich. Lipofectamine for siRNA transfection was purchased from Life Technologies. siRNA oligonucleotides and shRNA lentiviral particles were purchased from Santa Cruz. Dentin slices were purchased from Immunodiagnostic Systems.

### Cell lines and culture conditions

LNCaP and PC3 cells were purchased from the Korean Cell Line Bank (Seoul, Korea). Cell lines were validated by STR-PCR analysis by the Korean Cell Line Bank, and the STR profiles of each cell line can be accessed from their website (https://cellbank.snu.ac.kr/main/tmpl/sub_main.php?m_cd=22&m_id=0503). Cells were cultured in high-glucose DMEM (Lonza) supplemented with 10% FBS (Life Technologies) and 1% penicillin and streptomycin (WELGENE, Korea).

### Flow cytometry

mtPC3 cells were authenticated by labelling with PE-conjugated mouse anti-human β2-microglobulin (BD Pharmingen). Human β2-microglobulin is associated with the HLA Class I antigen complex. During flow cytometry analyses, mouse bone marrow cells served as a negative control and human embryonic kidney-293 cells were utilized as a positive control. Briefly, 1 × 10^5^ cells were incubated with the antibody (1:100) for half an hour and softly washed with ice-cold PBS three times before analysis using FACSCalibur (BD Science).

### Western blotting

Cells were washed with ice-cold PBS and lysed with RIPA buffer (10 mM Tris pH 7.2, 150 mM NaCl, 0.1% sodium dodecyl sulfate, 1% Triton X-100, 1% sodium deoxycholate, and 5 mM ethylenediaminetetraacetic acid). A standard protocol for Western blotting was performed with cell lysates.

### Cell proliferation assay

Cells were incubated at the indicated time with 10% CCK solution in cell culture medium for 1 h at 37 °C. Optical density was then measured with an ELISA reader (iMARK Microplate Absorbance Reader, Bio-Rad, Hercules, CA, USA) at 450 nm.

### Cell migration assay

The cell migration assay was performed using trans-well plates with 8.0-μm polycarbonate membranes (Corning). 1 × 10^5^ cancer cells were seeded onto the upper chamber. The cells were tested for the migration toward the lower chamber during stimulation by serum with or without S100A4 (2 μg/ml) or S100A4 neutralizing antibodies (4A, 30 μg/ml). After 16 h, migrated cells were fixed with fixing solution, followed by crystal violet staining for visualization with a microscope (Olympus BX51, 20x objective lenses, DP72 camera, DP2-BSW software (version 2.2)).

### Conditioned medium preparation

1 × 10^6^ cancer cells were seeded onto a 60-mm culture dish with DMEM and incubated overnight. The next day, the culture medium was exchanged to alpha-MEM (WELGENE) and further incubated for 24 h. Then, the supernatant was collected and centrifuged at 1200 rpm to remove dead cells. A 3:7 (supernatant to fresh medium) ratio was used unless specifically indicated in the figure legend.

### Osteoclast differentiation

BMMs were prepared from 5-week-old female ICR mice as previously described [29]. Pre-osteoclasts were generated by culturing BMMs with M-CSF (30 ng/mL) and RANKL (50 ng/mL) for 36 to 48 h. Then, mature osteoclast formation was induced by incubating pre-osteoclasts with M-CSF (30 ng/mL) and conditioned medium from cancer cells. Multinucleated TRAP+ cells usually formed within 24 to 48 h after conditioned medium treatment. TRAP+ cells with 3 or more nuclei were visualized under a microscope and considered to be mature osteoclasts (Olympus BX51, 20x objective lenses, DP72 camera, DP2-BSW software (version 2.2)).

### Real-time PCR analysis

A standard protocol for real-time PCR analysis was pursued for the quantification of mRNA levels. Primers for real-time PCR analyses are as follows: human *hprt* (hypoxanthine-guanine phosphoribosyltransferase) forward, 5′- accccacgaagtgttggata-3′; human *hprt* reverse, 5′- aagcagatggccacagaact-3′; human *s100a4* forward, 5′-gcccagcttcttggggaaaa-3′; human *s100a4* reverse, 5′- atggcgatgcaggacaggaa-3′.

### Enzyme-linked immunosorbent assay (ELISA)

1 × 10^5^ cancer cells/well were seeded onto a 48-well tissue culture plate and incubated overnight. The culture medium was replaced with 100 μL serum-free medium and further incubated for 24 h. Supernatant was collected and subjected to ELISA with a human S100A4 ELISA kit (CycLex Co.) according to the manufacturer’s protocol.

### Osteoclast resorption assay

A dentin slice was placed into each well of a 48-well tissue culture plate, after which 4 × 10^4^ BMMs were seeded as well. Osteoclast differentiation and bone resorption were induced by supplementing M-CSF (30 ng/mL) and RANKL (50 ng/mL). Dentin slices were washed with distilled water for cell removal and mounted on glass slides. The resorbed depth and area were calculated by inspecting the dentin surface using a Zeiss LSM 5 PASCAL laser-scanning microscope (20x objective lenses; Carl Zeiss Microimaging GmbH, Goettingen, Germany). The Zeiss LSM Image Browser program was utilized (version 3.0 SP3).

### Gene knockdown

S100A4 and control shRNA lentiviral particles were purchased from Santa Cruz Biotechnology, Inc. mtPC3 cells were transduced with lentiviral particles and incubated for 2 days. The cells were incubated with puromycin (10 μg/mL) for an additional 3 days to sort the successfully transduced cells. For siRNA transfection, BMMs were seeded in the presence of M-CSF (30 ng/mL) and incubated overnight. The next day, Lipofectamine 2000 (Invitrogen) was used for the formation of the liposome complex containing siRNAs. The complex was incubated with BMMs for 6 h, followed by fresh media replacement.

### GEPIA analysis

S100A4 expression patterns across various human cancer were analyzed using GEPIA, an online tool for The Cancer Genome Atlas (TCGA) and Gene Tissue Expression (GTEX) databases [30]. Furthermore, the GEPIA survival analysis was used to verify the relationship between S100A4 expression and prostate cancer prognosis. The correlation of gene expression was evaluated using Spearman’s correlation analysis.

### Statistics

Data are presented as the mean ± SD of biological replicates. An unpaired two-tailed Student’s *t*-test was used to define differences between two samples. One-way ANOVA or Two-way ANOVA with a post hoc Bonferroni or Tukey’s test were used for analyses of multiple groups. All statistical tests were performed using SigmaPlot 11.0 (Version 11.2.0.11, Systat software Inc., San Jose, CA, USA). *p* < 0.05 was considered statistically significant.

## Results

### S100A4 is up-regulated in bone-metastatic prostate cancer cells

**Fig. 1:**
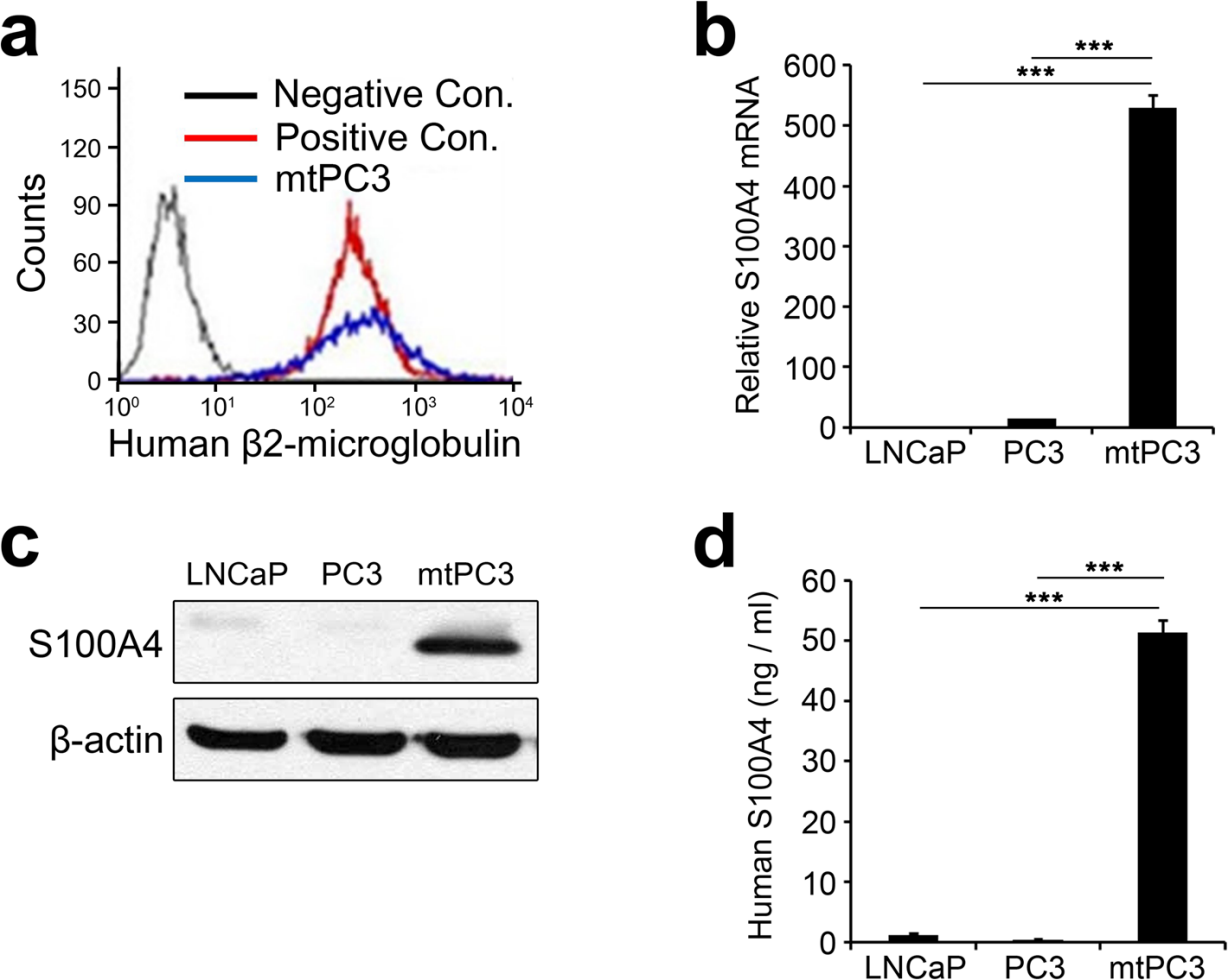
S100A4 expression and secretion is elevated in bone-metastatic prostate cancer cells. **a** Flow cytometry with anti-human β2-microglobulin Ab verified these bone-metastatic PC3 cells (mtPC3s) as human cells. Neg. Cont., mouse bone marrow cells; Pos. Cont., HEK-293 cells. **b** S100A4 mRNA expression in LNCaP, PC3, and mtPC3 cells was analyzed with real-time PCR. *n* = 3 per group. **c** S100A4 protein expression in LNCaP, PC3, and mtPC3 cells was analyzed by western-blotting. **d** Levels of S100A4 protein secretion in LNCaP, PC3, and mtPC3 cells in cell culture medium were measured by ELISA. *n* = 3 per group. ***p < 0.001 by one-way ANOVA with post hoc Tukey’s test. Data are presented as the mean ± SD. The full-length gels are presented in Supplementary Fig. 3a

To obtain bone-metastasized prostate cancer cells, we performed an in vivo selection experiment previously described for collecting highly bone-metastatic breast cancer cells [28]. Human prostate cancer PC3 cells were inoculated into the left ventricle of immune compromised Balb-c/nu mice, and 8 weeks later whole bone marrow cells were flushed from the long bones. Then, the cells were cultured for 2 months in vitro to remove mortal cells. The remaining cells were re-injected into mice. After 8 weeks, bone marrow cells were harvested and cultured again for 2 months. The cells obtained after the second round of selection were verified to be homogenous human cells by flow cytometry with anti-human β2-microglobulin antibody (Fig. 1a). We named this subline of PC3 cells mtPC3 cells (bone-metastasized PC3 cells). Next, we assessed the expression levels of S100A4 in the mtPC3 line. mtPC3 cells showed significantly higher mRNA expression levels of S100A4 compared to their parent cells (Fig. 1b). Western blotting of cell lysates revealed that the intracellular protein level of S100A4 was also markedly elevated in mtPC3 cells (Fig. 1c). We next tested whether the S100A4 protein was secreted from the cells by performing a human S100A4 ELISA with conditioned medium (CM) of cancer cells. While S100A4 was almost undetectable in LNCaP and PC3 cell CM, the concentration of S100A4 was as high as 51.4 ± 2.04 ng/ml in mtPC3 CM (Fig. 1d). Altogether, these results indicate that bone-metastatic prostate cancer cells express high levels of S100A4 mRNA and protein, and secrete a greatly elevated level of S100A4 protein into the extracellular milieu.

### Intracellular S100A4 increases cell proliferation of bone-metastatic prostate cancer

**Fig. 2:**
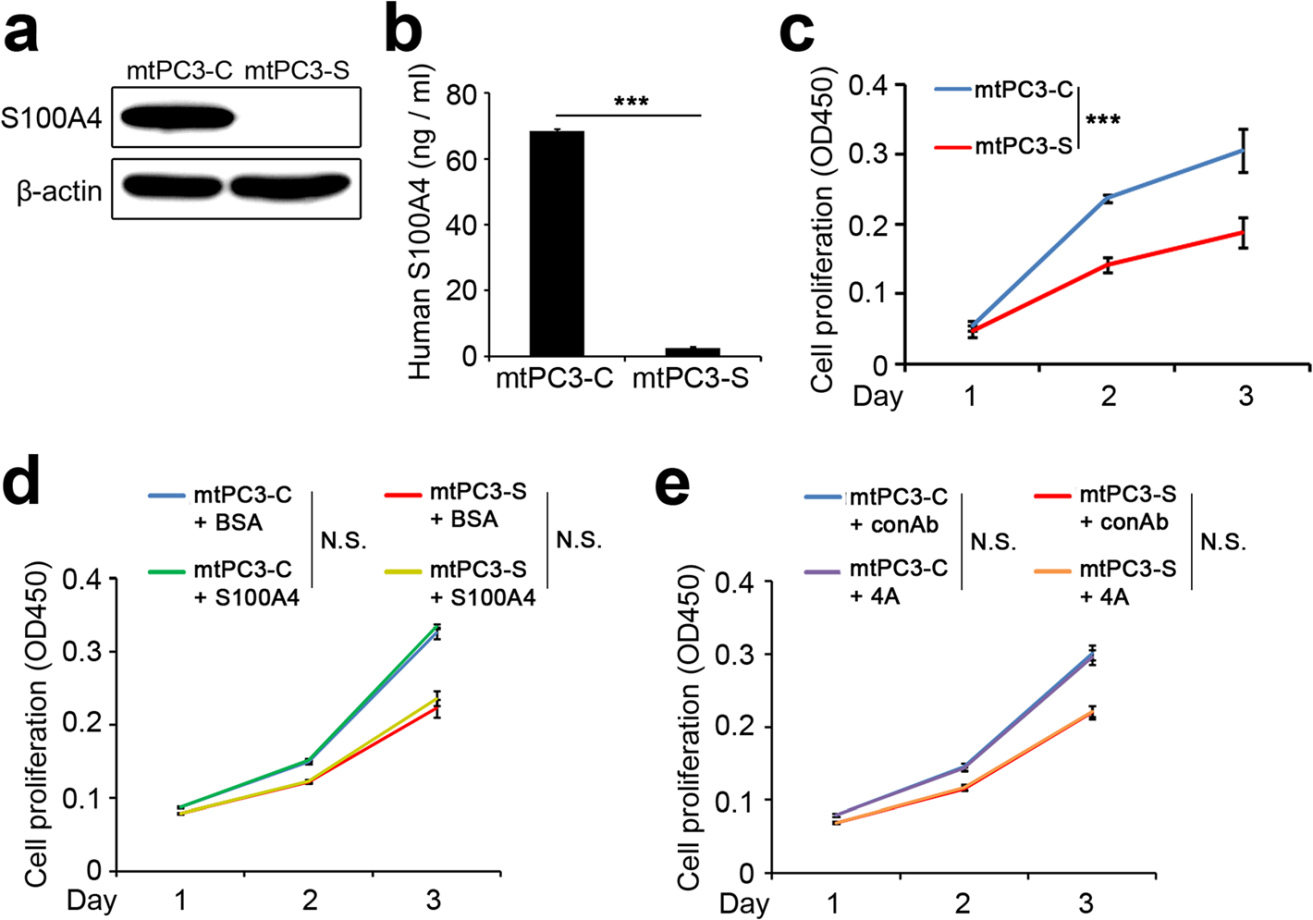
Intracellular S100A4 promotes proliferation of bone-metastatic prostate cancer cells. **a, b** Lentiviral transduction of S100A4 shRNA efficiently reduced S100A4 protein levels in mtPC3 cells (**a**) as well as S100A4 protein secretion in culture medium from mtPC3 cells (**b**). ***p < 0.001 by unpaired two-tailed Student’s *t*-test. **c-e** Proliferation of mtPC3 cells with control shRNA (mtPC3-C) vs. S100A4 shRNA (mtPC3-S) was analyzed with the CCK kit (**c**). Proliferation of mtPC3-C or mtPC3-S cells in the presence of BSA or recombinant human S100A4 (S100A4; 2 μg/ml) was analyzed with the CCK kit (**d**). Proliferation of mtPC3-C or mtPC3-S cells in the presence of control antibody or monoclonal anti-S100A4 antibody (4A; 30 μg/ml) was analyzed with the CCK kit (**e**). ***p < 0.001 by two-way ANOVA with post hoc Bonferroni’s test. N.S., not significant. Data are presented as the mean ± SD. The full-length gels are presented in Supplementary Fig. 3b

Previous reports have shown that S100A4 regulates cell growth in a context-dependent manner [31, 32]. Thus, we examined the potential role of S100A4 in the proliferation of mtPC3 cells utilizing lentiviral transduction of S100A4 shRNA. Western blotting of whole cell lysates (Fig. 2a) and ELISA of culture supernatants (Fig. 2b) demonstrated successful down-regulation of S100A4 by shRNA. The mtPC3 cells with S100A4 shRNA (mtPC3-S) showed significantly lower proliferation compared to mtPC3 cells with control shRNA (mtPC3-C) (Fig. 2c). We next tested whether the proliferation of mtPC3 cells was regulated by intracellular or secreted S100A4. Interestingly, recombinant human S100A4 (rhS100A4) did not affect the proliferation of either mtPC3-C or mtPC3-S cells (Fig. 2d). In addition, the proliferation of both mtPC3-C and mtPC3-S cells was not altered by treatment with an S100A4-neutralizing antibody, 4A [28] (Fig. 2e). The effect of extracellular S100A4 on proliferation was also not observed with LNCaP and PC3 cells (Supplementary Fig. 1). Taken together, these data indicate that S100A4 promotes proliferation of bone-metastatic prostate cancer cells by an intracellular mechanism.

### S100A4 promotes EMT traits and migration of prostate cancer cells

**Fig. 3:**
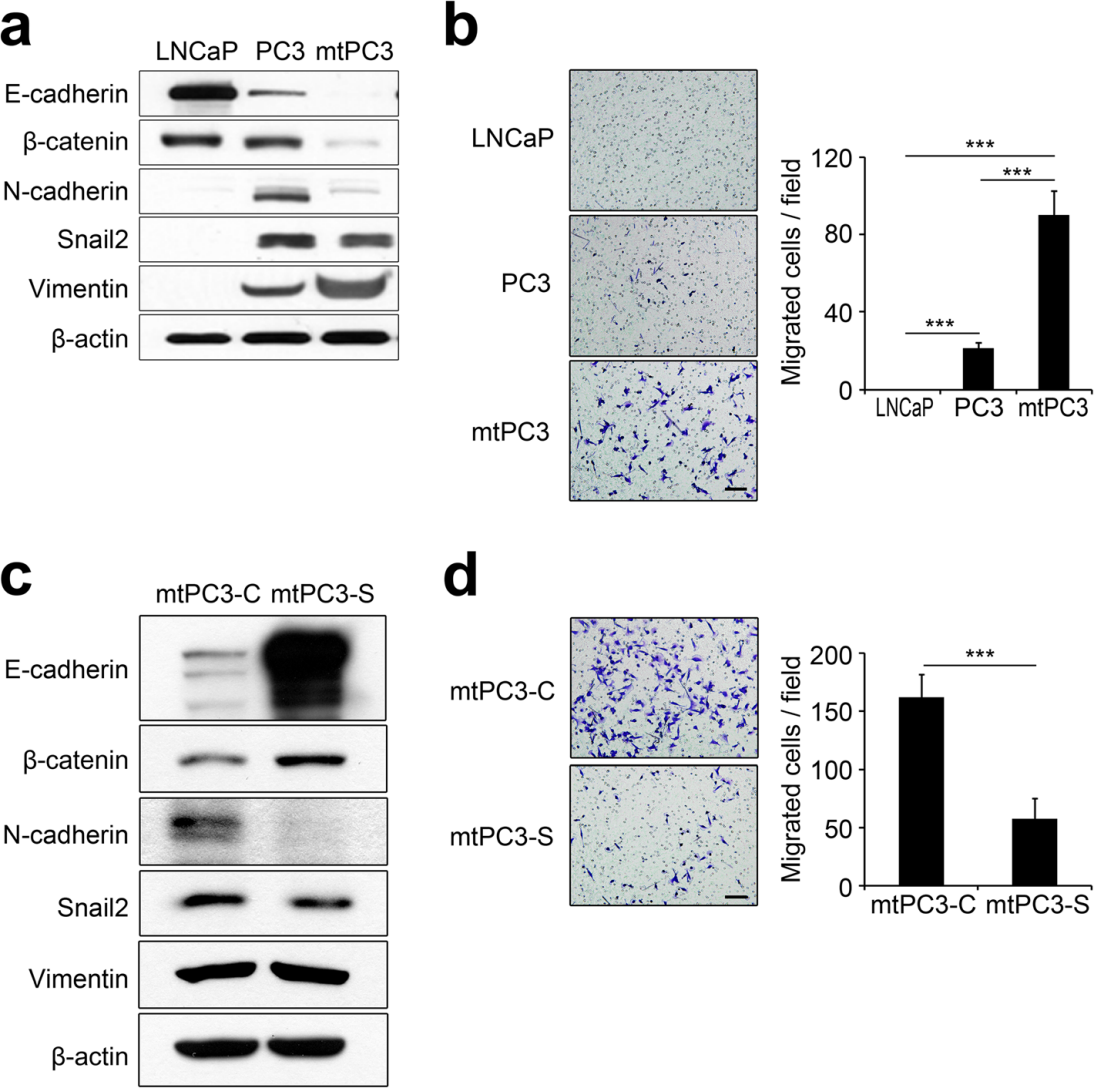
S100A4 promotes cell migration and EMT of bone-metastatic prostate cancer cells. **a** Protein levels of E-cadherin, β-catenin, N-cadherin, Snail2, Vimentin, and β-actin in LNCaP, PC3, and mtPC3 cells were determined by western blotting with the indicated antibodies. **b** Cell migration of LNCaP, PC3, and mtPC3 cells was measured by transwell assay. Representative images (left panels) and quantification of migrated cells (right panel). Scale bar = 100 μm. ***p < 0.001 by one-way ANOVA with post hoc Tukey’s test. **c** Protein levels of E-cadherin, β-catenin, N-cadherin, Snail2, Vimentin, and β-actin in mtPC3-C or mtPC3-S cells were determined by western blotting with the indicated antibodies. **d** Cell migration of mtPC3-C or mtPC3-S cells was measured by transwell assay. Representative images (left panels) and quantification of migrated cells (right panel). Scale bar = 100 μm. ***p < 0.001 by unpaired two-tailed Student’s *t*-test. Data are presented as the mean ± SD. The full-length gels are presented in Supplementary Fig. 3c and 3d

Since prostate cancer metastasis involves EMT [11, 12], we investigated whether mtPC3 cells underwent EMT by assessing protein levels of epithelial (E-cadherin and β-catenin) and mesenchymal (N-cadherin, snail2, and vimentin) markers. Western blot analysis demonstrated that the non-metastatic LNCaP prostate cancer cell line highly expressed E-cadherin and β-catenin, but not N-cadherin, snail2, or vimentin, indicating the epithelial characteristics of this cell line (Fig. 3a). PC3 cells expressed E-cadherin and β-catenin to a lesser extent than LNCaP and showed evident expression of N-cadherin, snail2, and vimentin, depicting both epithelial and mesenchymal characteristics (Fig. 3a). The mtPC3 cells showed a negligible amount of E-cadherin and β-catenin but had significant levels of vimentin, demonstrating the most mesenchymal characteristic among the three cell types tested (Fig. 3a). Consistent with mesenchymal characteristics, mtPC3 cells showed the highest cell migration capacity (Fig. 3b). Thus, we evaluated whether S100A4 affects cell migration and EMT. As shown in Fig. 3c, mtPC3-S cells showed significantly increased expression of E-cadherin and β-catenin and lower levels of N-cadherin compared to mtPC3-C cells (Fig. 3c). Although protein levels of vimentin and snail2 were not affected by S100A4 shRNA (Fig. 3c), these data indicate that mtPC3 cells lost some mesenchymal characteristics through S100A4 knock-down. Consistent with this proposition, mtPC3-S cells showed decreased cell migration compared to mtPC3-C cells (Fig. 3d).

**Fig. 4:**
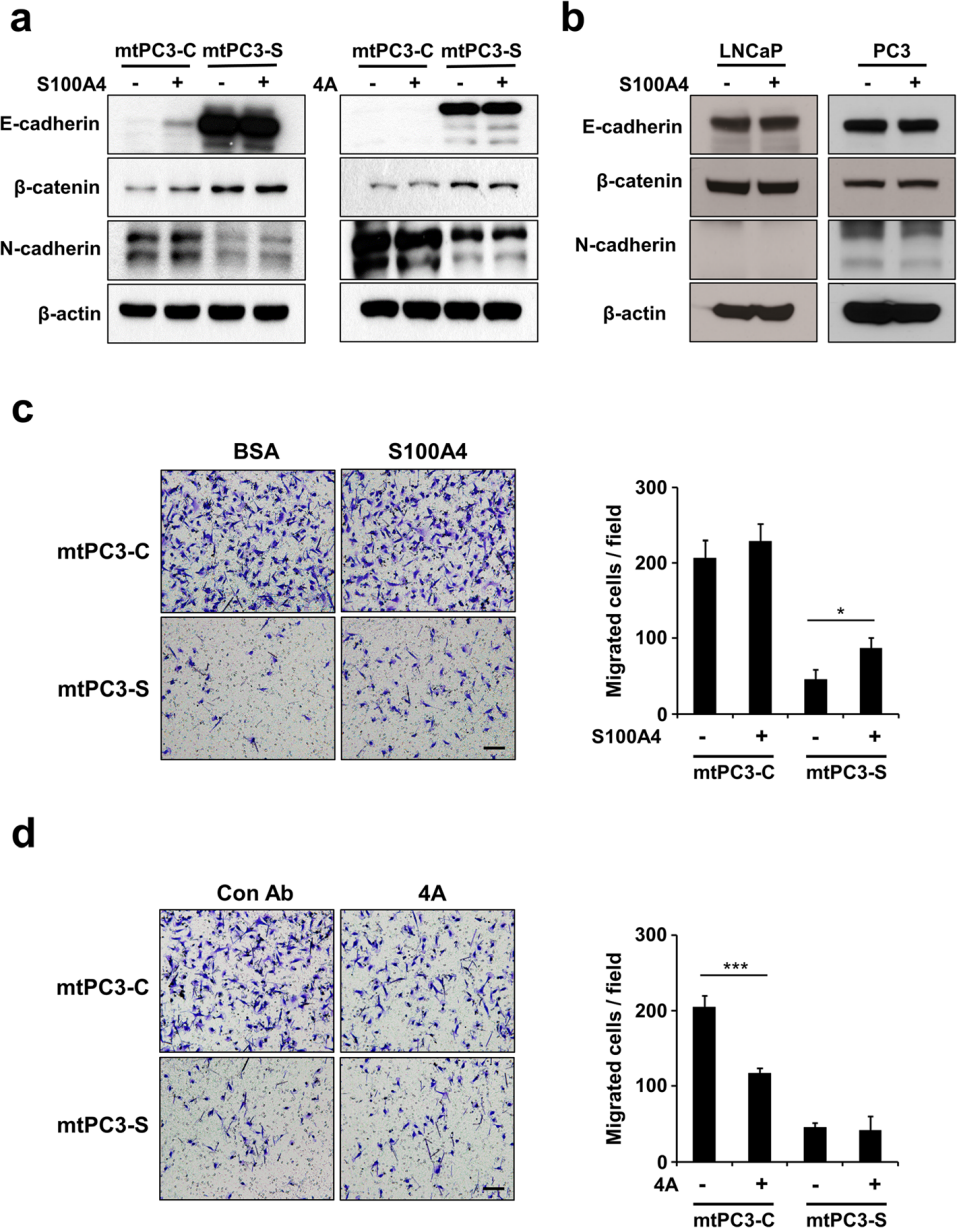
S100A4 promotes EMT and cell migration of bone-metastatic prostate cancer cells. **a,** mtPC3-C or mtPC3-S cells were cultured in the presence of BSA, S100A4 (2 μg/ml), control antibody, or 4A (30 μg/ml) for 48 h, and then protein levels of E-cadherin, β-catenin, N-cadherin, and β-actin were determined by western blotting with the indicated antibodies. **b** The protein levels of E-cadherin, β-catenin and N-cadherin were analyzed by western blotting with lysates from LNCaP and PC3 cells cultured in the presence of BSA or S100A4 (2 μg/ml) for 48 h. **c** Cell migration of mtPC3-C or mtPC3-S cells was measured by transwell assay in the presence of BSA or S100A4 (2 μg/ml). Representative images (left panels) and quantification of migrated cells (right panel). Scale bar = 100 μm. **d** Cell migration of mtPC3-C or mtPC3-S cells was measured by transwell assay in the presence of control antibody or 4A (30 μg/ml). Representative images (left panels) and quantification of migrated cells (right panel). Scale bar = 100 μm. *p < 0.05 and ***p < 0.001 by one-way ANOVA with post hoc Tukey’s test. Data are presented as the mean ± SD. The full-length gels are presented in Supplementary Fig. 3e and 3 f

As the effect of S100A4 knock-down could be attributed to altered levels of intracellular and/or extracellular S100A4, we next sought to dissect the role of intracellular versus extracellular S100A4 on EMT and migration in mtPC3 cells. To evaluate the function of extracellular S100A4, we treated mtPC3-C and mtPC3-S cells with rhS100A4 and assessed the levels of EMT marker proteins. rhS100A4 did not change the expression levels of E-cadherin, β-catenin, or N-cadherin in mtPC3-S cells; however, it slightly increased E-cadherin and β-catenin expression in mtPC3-C cells (Fig. 4a). In addition, the S100A4-neutralizing antibody 4A did not alter E-cadherin, β-catenin, or N-cadherin expression in mtPC3-C or mtPC3-S cells (Fig. 4a). As the mtPC3 cells had already undergone EMT and possessed mesenchymal properties, it is possible that exogenous treatment of rhS100A4 or neutralizing antibody does not affect mtPC3 characteristics, even if EMT of mtPC3 cells were driven by extracellular S100A4. Thus, we investigated whether rhS100A4 influences EMT of LNCaP and PC3 cells which have relatively more epithelial properties. The levels of EMT markers were not changed by rhS100A4 in both cells (Fig. 4b). These results indicate that EMT in mtPC3 cells is regulated by intracellular, not extracellular, S100A4. Interestingly, cell migration of mtPC3-S cells was partially rescued by rhS100A4 (Fig. 4c), and 4A decreased mtPC3-C cells migration (Fig. 4d). Taken together, these data suggest that EMT of mtPC3 cells is enhanced by intracellular S100A4, while cell migration is promoted by both intracellular and extracellular S100A4.

### S100A4 mediates bone-metastatic prostate cancer-induced osteoclastogenesis via RAGE

**Fig. 5:**
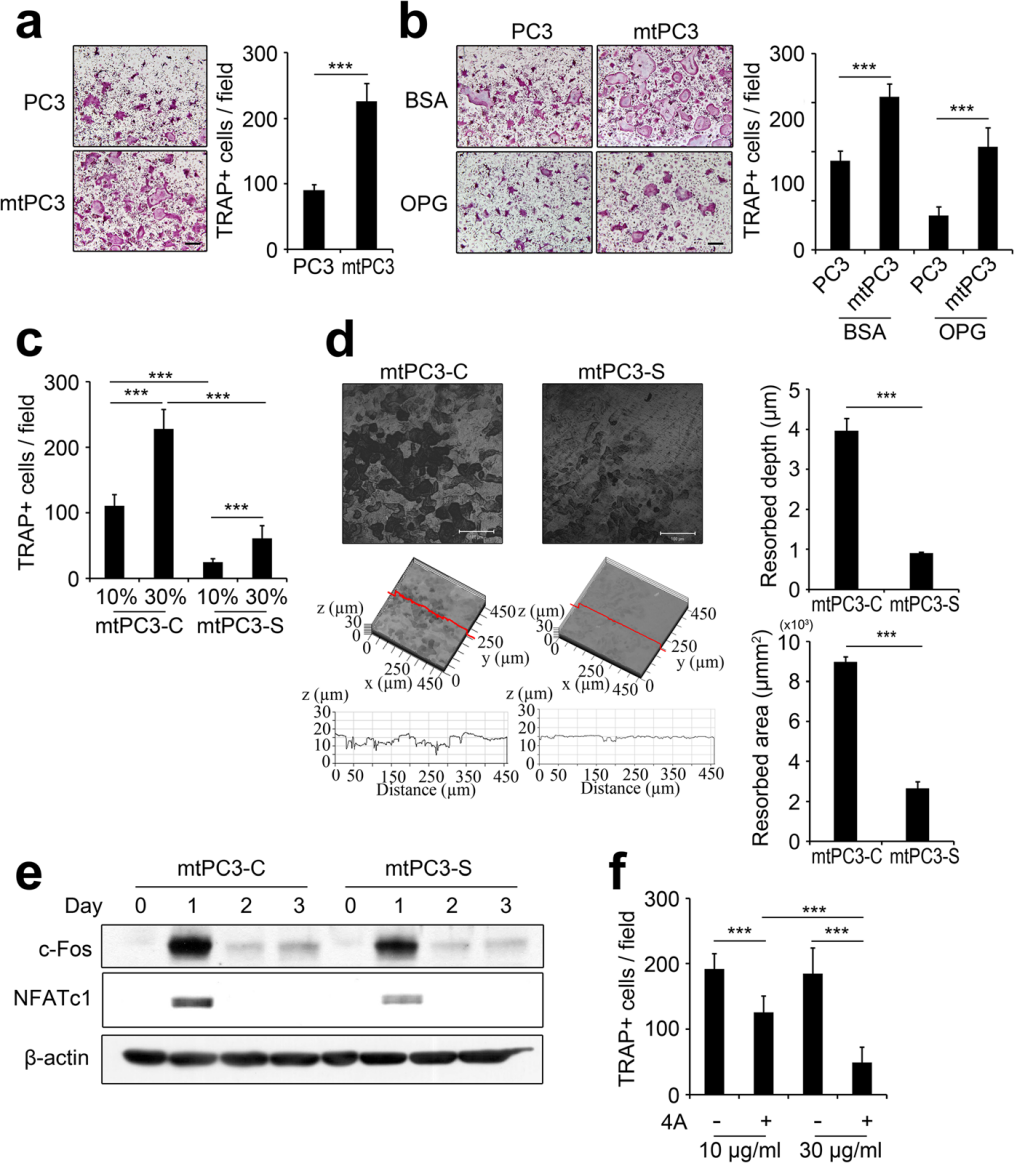
Bone-metastatic prostate cancer cells promote osteoclastogenesis by secretion of S100A4**. a** Pre-osteoclasts (pOCs) received PC3- or mtPC3-CM and were then tested for osteoclast generation. Representative tartrate-resistant acid phosphatase (TRAP)-stained images (left panels) and quantification of TRAP+ multinucleated cells (MNCs) with 3 or more nuclei (right panel). Scale bar = 100 μm. ***p < 0.001 by unpaired two-tailed Student’s *t*-test. **b** pOCs were treated with 30% PC3-CM or mtPC3-CM with BSA or OPG (100 ng/mL). Representative TRAP-stained images (left panel) and quantification of TRAP+ MNCs (right panel). Scale bar = 100 μm. ***p < 0.001 by one-way ANOVA with post hoc Tukey’s test. **c** pOCs receiving the indicated percentages of CM from mtPC3-C or mtPC3-S cells were TRAP-stained and then counted for TRAP+ MNCs. ***p < 0.001 by one-way ANOVA with post hoc Tukey’s test. **d** pOCs receiving 30% mtPC3-C-CM or mtPC3-S-CM were cultured on dentin slices. Representative confocal images of dentin surfaces (left panels) and values of pit depth and resorbed area (right panels) are presented. Scale bar = 100 μm. ***p < 0.001 by unpaired two-tailed Student’s *t*-test. **e** Western blots of c-Fos, NFATc1, and β-actin in BMM and pOCs after the indicated number of days with 30% mtPC3-C-CM or mtPC3-S-CM. **f** pOCs were tested with 30% mtPC3-CM, together with the indicated concentrations of control antibody or 4A. TRAP+ MNCs were counted. ***p < 0.001 by one-way ANOVA with post hoc Tukey’s test. Data were presented as the mean ± SD. The full-length gels are presented in Supplementary Fig. 3 g

We next investigated whether S100A4 secreted from mtPC3 cells affects osteolysis. We used mouse primary bone marrow-derived macrophages (BMMs) for osteoclastogenesis. CM from PC3 and mtPC3 cells were applied to pre-osteoclasts (pOCs) generated by priming BMMs with RANKL. The mtPC3 CM enhanced the number of mature tartrate-resistant acid phosphatase-positive (TRAP^+^) multinucleated cells (osteoclasts; OCs) compared with the CM from PC3 cells (Fig. 5a). Because RANKL is an essential factor for osteoclastogenesis, we tested whether osteoprotegerin (OPG, an RANKL antagonist) could inhibit the effect of mtPC3 CM. Surprisingly, OPG treatment only partially reduced osteoclastogenesis, suggesting that RANKL was not the sole factor triggering osteoclastogenesis in mtPC3 CM (Fig. 5b). We next compared the effect of CM from mtPC3-C and mtPC3-S cells to evaluate the role of secreted S100A4 in stimulating osteoclastogenesis. As shown in Fig. 5c and d, CM from mtPC3-C cells successfully induced differentiation and resorption activity of OCs (Fig. 5c and d). However, the CM from mtPC3-S cells only weakly induced osteoclast formation and bone resorption (Fig. 5c and d). Protein levels of c-Fos and NFATc1, key transcription factors for osteoclastogenesis, were consistently lower in pOCs treated with CM from mtPC3-S cells compared to those treated with CM from mtPC3-C cells (Fig. 5e). Moreover, neutralization of S100A4 with 4A Ab inhibited mtPC3 CM-induced osteoclastogenesis in a dose-dependent manner (Fig. 5f). Collectively, these results suggest that bone-metastatic prostate cancer-derived S100A4 stimulates osteoclastogenesis.

**Fig. 6:**
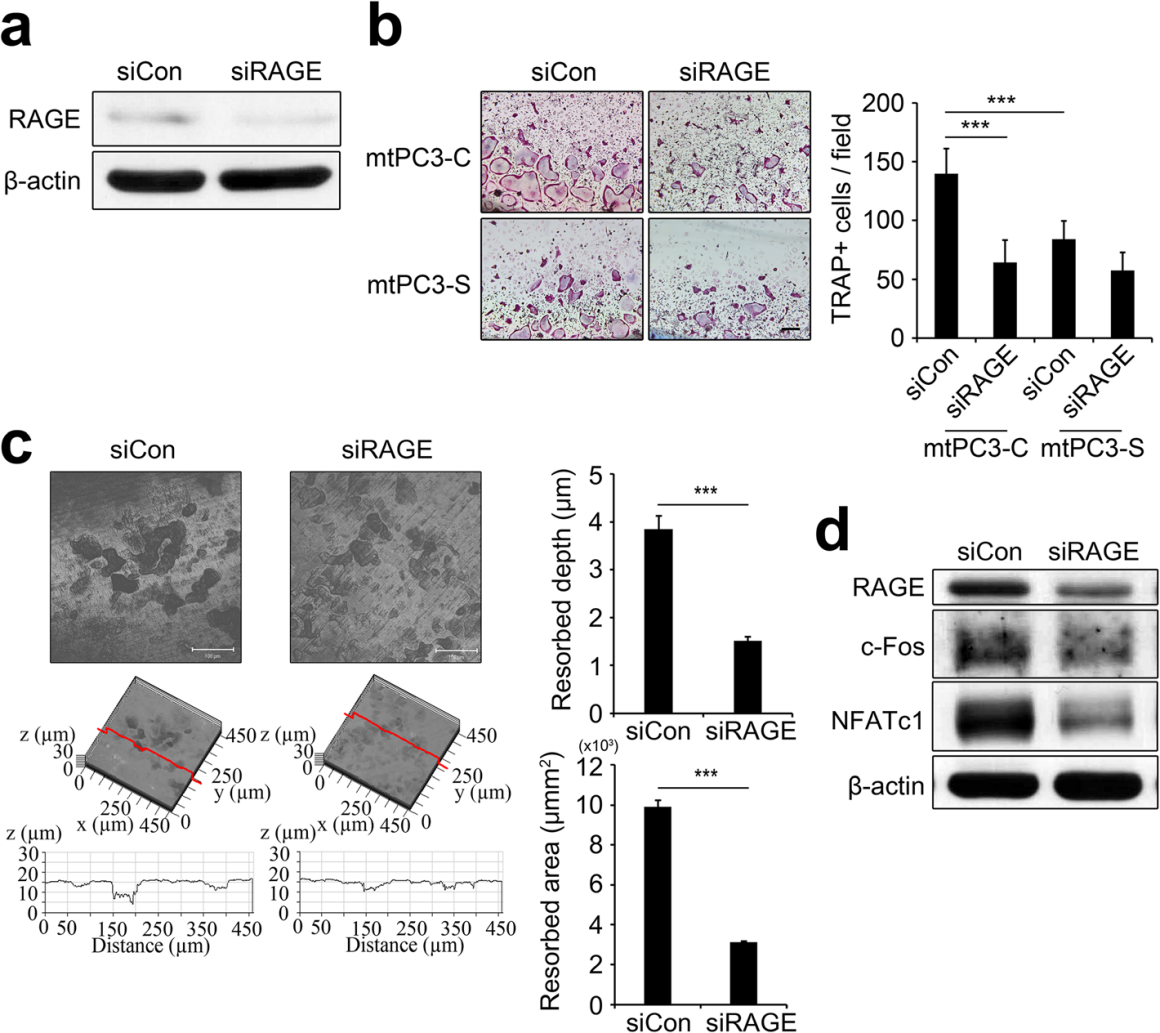
mtPC3-CM induced osteoclastogenesis via RAGE. **a** Control (siCon) or RAGE siRNA (siRAGE) transfected BMMs were subjected to Western blotting to assess protein levels of RAGE and β-actin. **b** pOCs transfected with siCon or siRAGE were treated with 30% mtPC3-C-CM or mtPC3-S-CM for osteoclastogenesis. Representative TRAP-stained images (left panels) and quantification of TRAP+ MNCs (right panel). Scale bar = 100 μm. ***p < 0.001 by one-way ANOVA with post hoc Tukey’s test. **c** pOCs transfected with siCon or siRAGE were cultured on dentin slices with 30% mtPC3-CM. Representative confocal images of dentine surfaces (left panels) and values of the depth of pits and resorbed area (right panels) are presented. Scale bar = 100 μm. ***p < 0.001 by unpaired two-tailed Student’s *t*-test. **d** pOCs transfected with siCon or siRAGE were treated with 30% mtPC3-CM for 24 h and were then subjected to western blotting to assess the protein levels of RAGE, c-Fos, NFATc1, and β-actin. Data are presented as the mean ± SD. The full-length gels are presented in Supplementary Fig. 3 h and 3i

S100A4 protein has been shown to bind the cell surface receptor RAGE, mediating tumor cell survival and metastasis [16]. Our previous study on bone-metastatic breast cancer showed that RAGE plays a crucial role in S100A4-stimulated osteoclastogenesis [28]. Therefore, we assessed whether bone-metastatic prostate cancer also utilizes RAGE for osteoclast development. A loss of function approach using RAGE-targeting siRNA led to successful down-regulation of RAGE in BMMs at the protein level (Fig. 6a). RAGE knock-down significantly reduced osteoclast differentiation in cells treated with mtPC3-C-CM, while osteoclast formation with mtPC3-S-CM was not significantly different between the control knock-down and RAGE knock-down groups (Fig. 6b). In addition, bone resorption in mtPC3-CM-treated culture was significantly decreased by RAGE knock-down (Fig. 6c). Protein expression of c-Fos and NFATc1 upregulated by mtPC3-CM were consistently attenuated by RAGE knock-down (Fig. 6d). Taken together, our data show that S100A4 secreted from bone-metastatic prostate cancer interacts with RAGE on osteoclast precursor cells to accelerate osteoclast development.

## Discussion

In this study, we found that S100A4 is up-regulated in bone-metastatic prostate cancer cells, the mtPC3 cells (Fig. 1). We observed that S100A4-high mtPC3 cells exhibited more mesenchymal characteristics than their parent cells (Fig. 3). This mesenchymal phenotype was decreased by S100A4 knock-down, but was not altered by recombinant S100A4 or S100A4-neutralizing antibodies (Fig. 4). Cell proliferation of mtPC3 was also decreased by S100A4 knock-down, but not by exogenous S100A4 treatment or S100A4 neutralization (Fig. 2). Moreover, we found that mtPC3-CM stimulated osteoclastogenesis, which was attributed to elevated secretion of S100A4 from mtPC3 cells (Fig. 5). Similarly, shRNA-mediated knockdown or administration of S100A4-neutralizing antibody nullified the enhanced osteoclastogenesis induced by mtPC3-CM (Fig. 5). Furthermore, we observed that RAGE on the surface of pre-osteoclasts was responsible for the mtPC3-derived S100A4-mediated enhancement of osteoclastogenesis (Fig. 6). Altogether, we suggest that elevated expression of S100A4 in bone-metastatic prostate cancers contributes to cancer proliferation, migration, and EMT by intra- and/or extracellular mechanisms. After bone-metastasis, secreted S100A4 from metastatic-prostate cancer cells may promote osteoclastogenesis via RAGE, leading to bone destruction.

The overall process of cancer metastasis is complicated, and various cellular events occur during this process. At the early stage of metastasis, epithelial tumor cells acquire the ability to detach from the primary tumor mass and invade through the basement membrane. These tumor cells then intravasate into the circulatory system, extravasate into a preferred distant site depending on the type of tumor, and interact with the metastatic environment [33, 34]. EMT is considered to be the most critical initiation step in this process. The clinical correlation between EMT and poor survival implies the importance of EMT [35]. Loss of epithelial properties in cancer cells by EMT not only causes cell detachment from the primary tumor mass, promoting intravasation, but also enhances angiogenesis, supporting the dispersion of tumor cells through the systemic circulation [36].

It has been generally appreciated that E-cadherin and N-cadherin show an opposite expression pattern [8]. In our study, mtPC3 cells showed no increase in N-cadherin and snail2 while obtaining mesenchymal properties represented by reduction of E-cadherin and elevation of vimentin. Recently a study reported the cases of no correlation between snail2 expression and E-cadherin loss [37]. This report and our data suggest a possibility that the pattern of EMT marker expression deviates from the typical one in certain circumstances, for example bone metastasis. The pathophysiological significance of this interesting phenomenon should be further explored. Although mtPC3 cells expressed less N-cadherin and snail2 than their parent cells, our overall data indicate that mtPC3 cells manifest mesenchymal characteristics considering the changes in other EMT markers (E-cadherin, β-catenin, and vimentin) and the increased migration.

Accumulating evidence supports the role of S100A4 as a crucial determinant of mesenchymal transition in various types of cancer cells. In glioma stem cells, S100A4-positive cells had tumor-initiating and sphere-forming properties [38]. S100A4 was found to function upstream of master EMT regulators such as Snail2 in glioblastoma [38]. The role of S100A4 in EMT is supported by RNA interference of S100A4 that suppressed the EMT process in endometrial cancer cells [39]. Moreover, down-regulation of S100A4 in breast cancer cells decreased EMT by suppressing the expression of MMP2 [40]. However, its role in prostate cancer has not been clearly studied yet. A previous study reported a correlation between EMT in prostate cancer cells and increased expression of S100A4 [41]. In our hands, down-regulation of S100A4 in mtPC3 cells restored E-cadherin and β-catenin proteins, and decreased N-cadherin and snail2 (Fig. 3). In an effort to evaluate the relevance of our results to human data, we analyzed the publicly available data set using the GEPIA platform. In this analysis, S100A4 was found to be expressed in various cancer types including prostate cancer (Supplementary Fig. 2a). In addition, the survival analysis revealed that patients with higher S100A4 expression levels tend to have poorer overall survival (Supplementary Fig. 2b). In line with this survival data, it was also reported that S100A4 level was significantly higher in prostate adenocarcinoma compared with benign prostate hyperplasia [42, 43]. These data may support our finding that S100A4 plays a role in EMT of prostate cancer cells increasing migration potency. While further examination is required to identify the specific mechanisms of EMT by S100A4 in bone-metastatic prostate cancer cells, our results demonstrate that S100A4 contributes to the EMT process in prostate cancer.

In our study, the alterations in epithelial and mesenchymal marker proteins by S100A4 knock-down were not observed with exogenous S100A4 treatment or with S100A4 neutralization (Fig. 4), suggesting that intracellular, not extracellular, S100A4 promotes mesenchymal transition of prostate tumor cells. Previous studies have demonstrated that S100A4 interacts with non-muscle myosin II, liprin, ezrin, and p53 in the intracellular microenvironment [18–21]. Among these S100A4-interacting proteins, p53 regulates EGF-mediated MMP2 transcription, which promotes EMT in breast cancer cells [40, 44]. Beach et al. have shown that phosphorylation and isoform switching of myosin II are elevated during EMT [45]. Yamasaki et al. and Chen et al. have shown that liprin and ezrin, respectively, were also involved in EMT [46, 47]. Thus, there is a possibility that intracellular S100A4 promotes EMT by interacting with these molecules in mtPC3 cells. More detailed mechanisms by which S100A4 mediates EMT in prostate cancers warrant further investigation.

Some proteins have dual functions in intracellular and extracellular microenvironments. For example, syntaxin2 regulates vesicle trafficking and cytokinesis in the intracellular space, while it acts as a morphogen after secretion [48–50]. Extracellular HMGB1 regulates inflammation as a damage-associated molecular pattern, while intracellular HMGB1 binds to DNA in the nucleus [51, 52]. Similarly, S100A4 also has dual intra- and extracellular functions. S100A4 regulates cellular proliferation, migration, invasion, apoptosis, and EMT in various tumor cells. However, there have been no reports dissecting the intra- and extracellular roles of S100A4 in tumor cells. In this study, we divided the functional roles of intra- and extracellular S100A4 in bone-metastatic prostate cancer cells using S100A4 knock-down, recombinant S100A4, and S100A4-neutralizing antibody. In mtPC3 cells, intracellular S100A4 mediates proliferation and EMT, while extracellular S100A4 contributes to osteoclastogenesis. Cell migration of mtPC3 cells was regulated by both intra- and extracellular S100A4.

In bone metastasis, the interaction between cancer cells and the bone marrow niche is crucial factor determining metastatic bone phenotype such as osteolytic, osteosclerotic, or mixed. Typically, prostate cancer patients with bone metastasis show an osteosclerotic bone phenotype. However, several studies have shown that factors such as MMP-7, Runx2, and CTGF can alter osteosclerotic bone metastasis to an osteolytic phenotype in prostate cancers [53–55]. We found that osteoclastogenesis was augmented by extracellular S100A4 secreted from mtPC3 cells (Fig. 5). The osteoclastogenesis-promoting effect of mtPC3 could potentially lead to osteolysis by prostate cancer cells metastasized to bone. However, whether S100A4 up-regulation can alter the in vivo bone phenotype by bone metastasis of prostate cancer cells should be demonstrated in further studies.

To our knowledge, this is the first study to dissect the intra- and extracellular functions of S100A4 in bone-metastatic prostate cancer. Given our previous findings regarding the inhibition of osteoblast matrix mineralization by recombinant S100A4 treatment [56] and on the elevated secretion of S100A4 from bone-metastatic breast cancer cells [28], we propose that ways to inhibit the function of S100A4 will lead to the prevention of bone loss in osteolytic bone metastases. Moreover, as the elevated expression of S100A4 has been implicated in the pathology of tissue fibrosis, rheumatoid arthritis and other types of cancer, a wide application of therapeutics against S100A4 may be expected.

## Conclusions

Our study demonstrates that S100A4, highly expressed in bone-metastatic prostate cancer cells, has dual intra- and extracellular functions. S100A4 promotes proliferation and EMT by intracellular mechanisms and, after secretion, activates osteoclastogenesis as a ligand for the cell surface receptor RAGE on osteoclast precursor cells. Theses combined effects of S100A4 may be crucial for osteolytic bone metastasis.

## Supplementary Information

**Additional file 1: Supplementary Fig. 1.** The effect of extracellular S100A4 on LNCaP and PC3 cells. Proliferation of LNCaP (a) and PC3 (b) cells in the presence of BSA or recombinant human S100A4 (S100A4; 2 μg/ml), was analyzed with the CCK kit.

**Additional file 2: Supplementary Fig. 2.** Survey of S100A4 expression in various cancer types and relationship with patient prognosis. Based on GEPIA online database, comparison of S100A4 expression in multiple human cancers including prostate cancer (a) and percent of overall survival rate according to the expression of S100A4 in prostate cancer (b) were analyzed.

**Additional file 3: Supplementary Fig. 3.** The uncropped full-length western blotting images of figures.

## Data Availability

Data supporting the results in the article are available from the corresponding author upon reasonable request.

## Abbreviations

BMM: Bone marrow-derived macrophage
CM: Conditioned medium;
ELISA: Enzyme-linked immunosorbent assay
EMT: Epithelial-mesenchymal transition
IL-8: Interleukin 8
OC: Osteoclast
OPG: Osteoprotegerin
PC: Prostate cancer
PTHrP: Parathyroid hormone-related peptide
RAGE: Receptor for advanced glycation end products
S100A4: S100 calcium-binding protein A4
TGF-β: Transforming growth factor-β
TRAP: Tartrate-resistant acid phosphatase.
