# Supplementary figures and images for: The role of S100A4 for bone metastasis in prostate cancer cells
Source: BMC Cancer. 2021 Feb 6;21:137. doi: 10.1186/s12885-021-07850-4 (PMC7868026; doi:10.1186/s12885-021-07850-4)

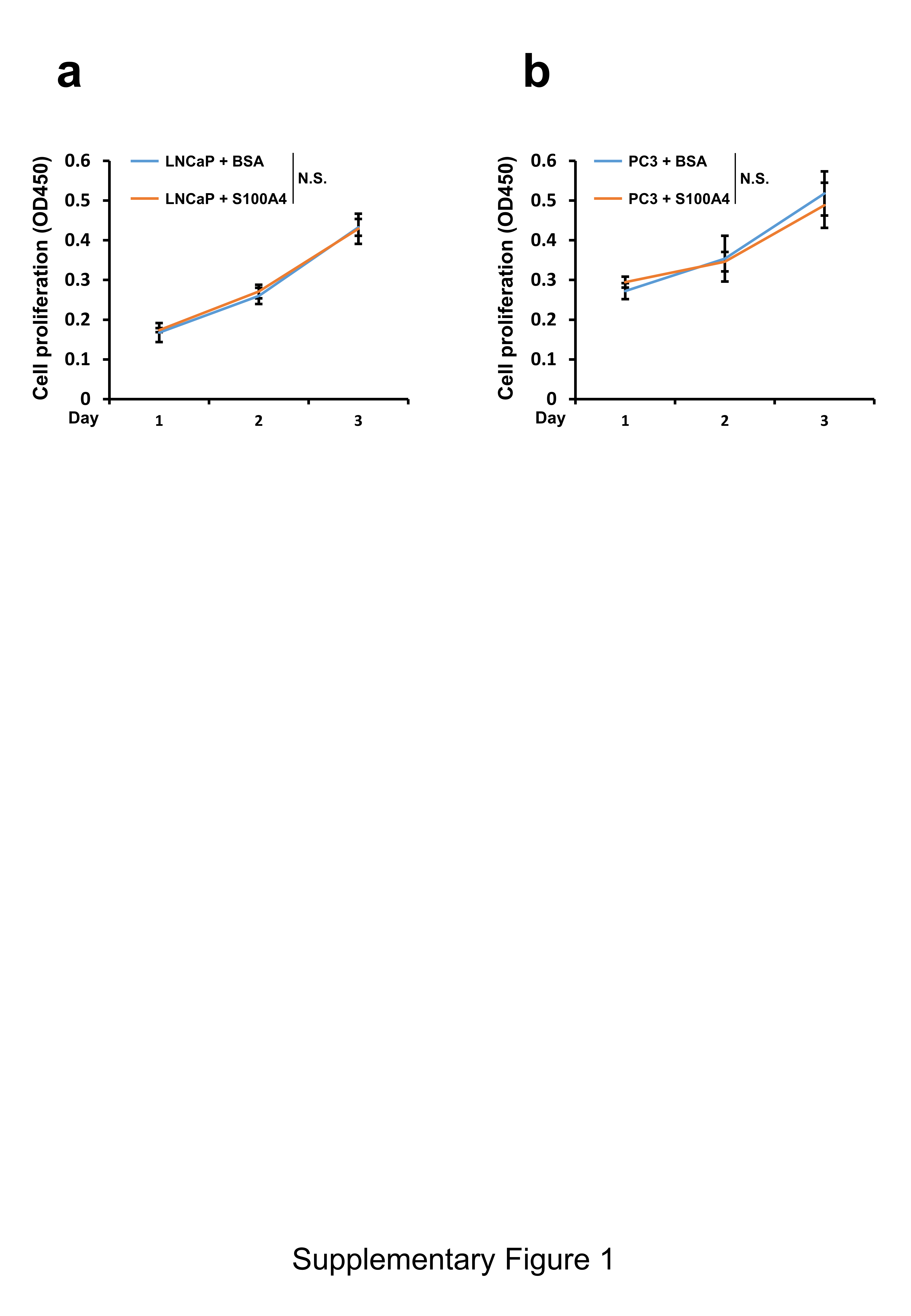

Supplement: Supplementary file 1 — Additional file 1: Supplementary Fig. 1. The effect of extracellular S100A4 on LNCaP and PC3 cells. Proliferation of LNCaP (a) and PC3 (b) cells in the presence of BSA or recombinant human S100A4 (S100A4; 2 μg/ml), was analyzed with the CCK kit. [file 12885_2021_7850_MOESM1_ESM.tif]

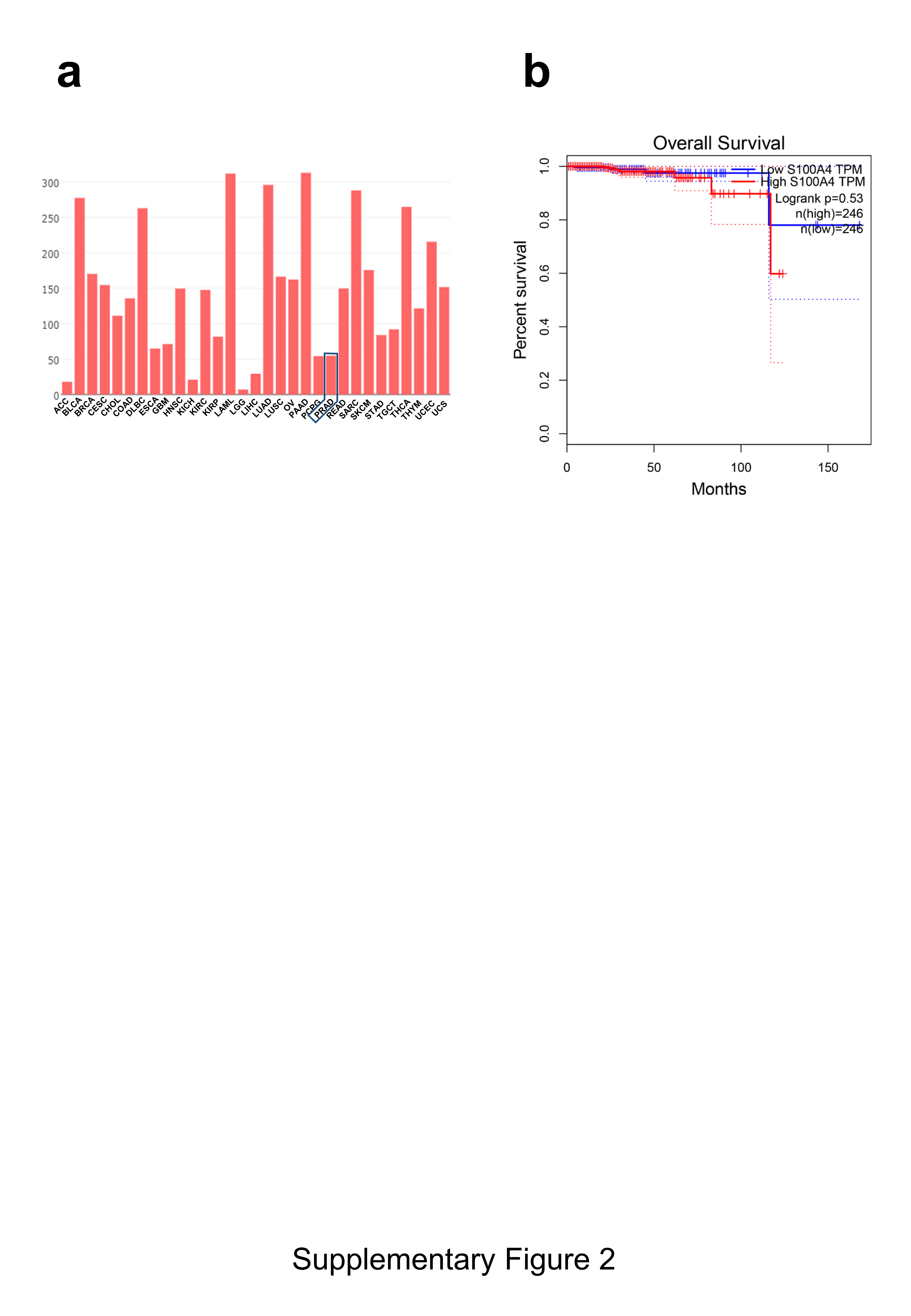

Supplement: Supplementary file 2 — Additional file 2: Supplementary Fig. 2. Survey of S100A4 expression in various cancer types and relationship with patient prognosis. Based on GEPIA online database, comparison of S100A4 expression in multiple human cancers including prostate cancer (a) and percent of overall survival rate according to the expression of S100A4 in prostate cancer (b) were analyzed. [file 12885_2021_7850_MOESM2_ESM.tif]
