# Supplementary material for: The role of S100A4 for bone metastasis in prostate cancer cells
Source: BMC Cancer. 2021 Feb 6;21:137. doi: 10.1186/s12885-021-07850-4 (PMC7868026; doi:10.1186/s12885-021-07850-4)

a Figure 1c uncropped images

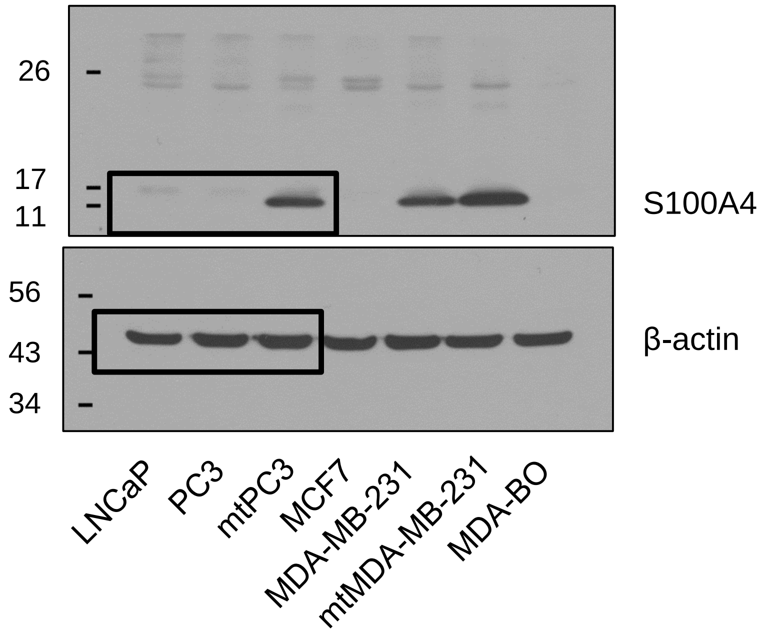

b Figure 2a uncropped images

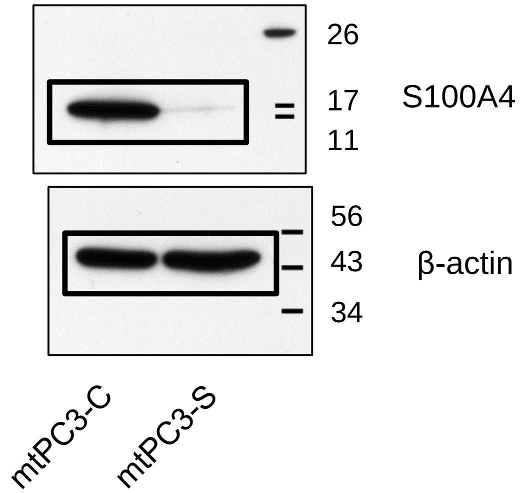

c Figure 3a uncropped images

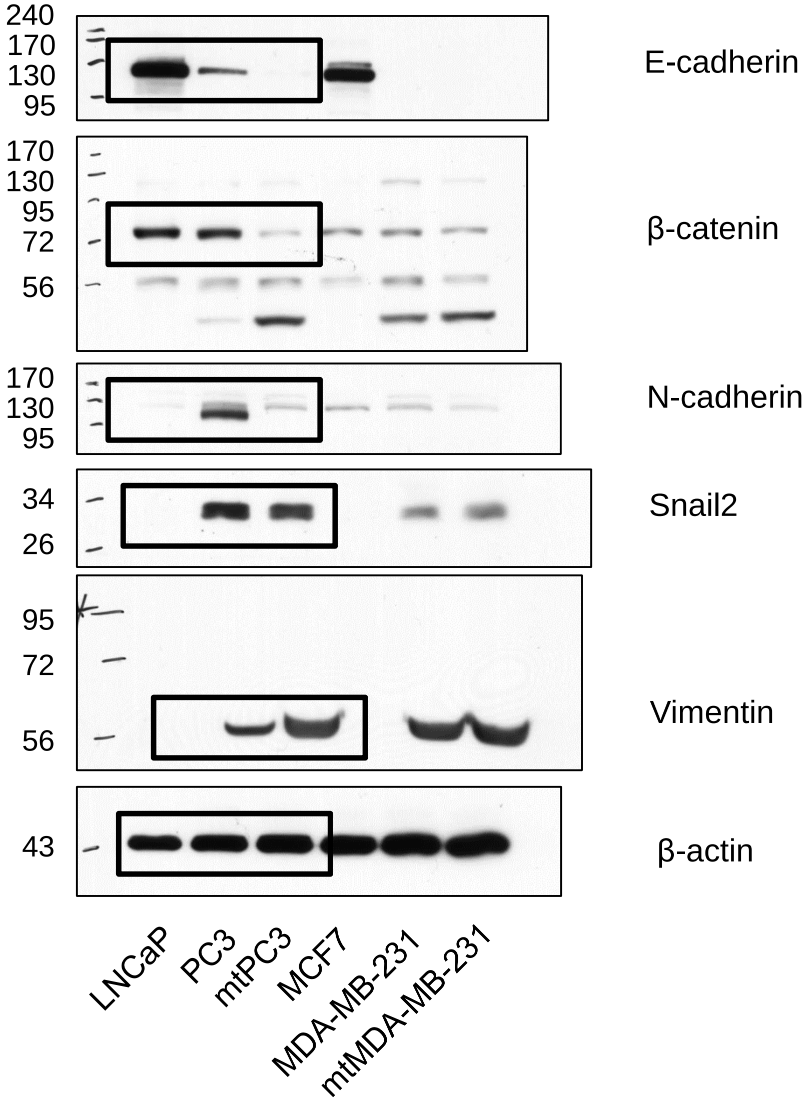

d Figure 3c uncropped images

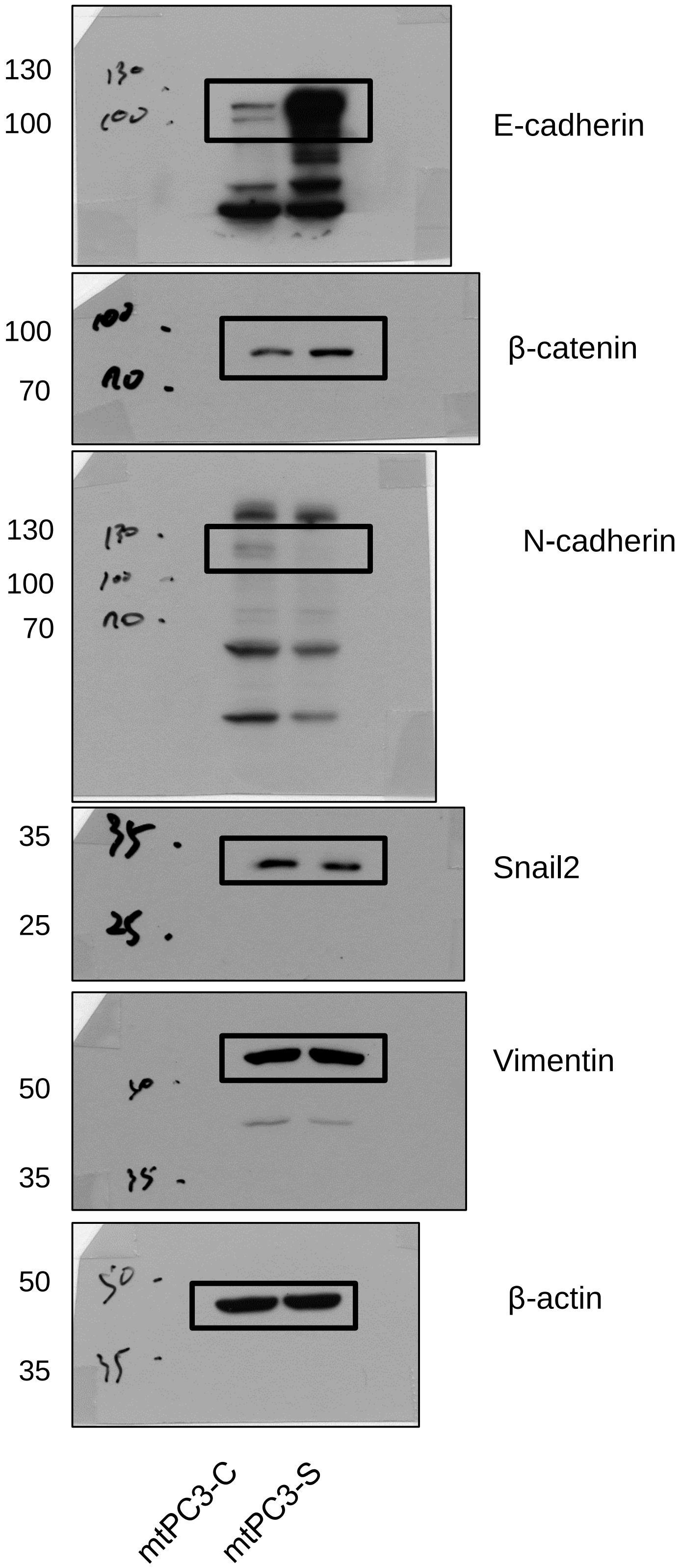

e Figure 4a uncropped images

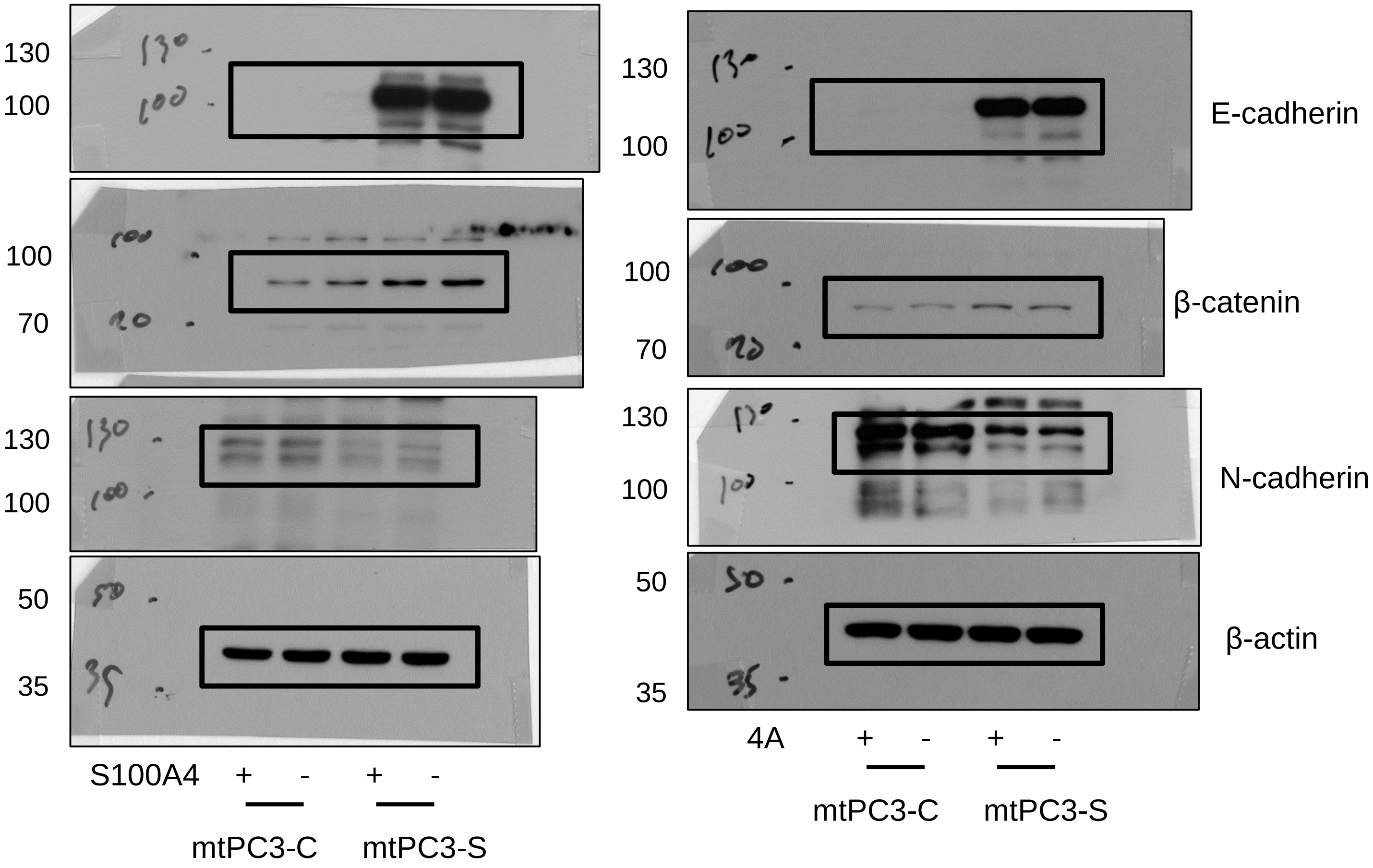

f Figure 4b uncropped images

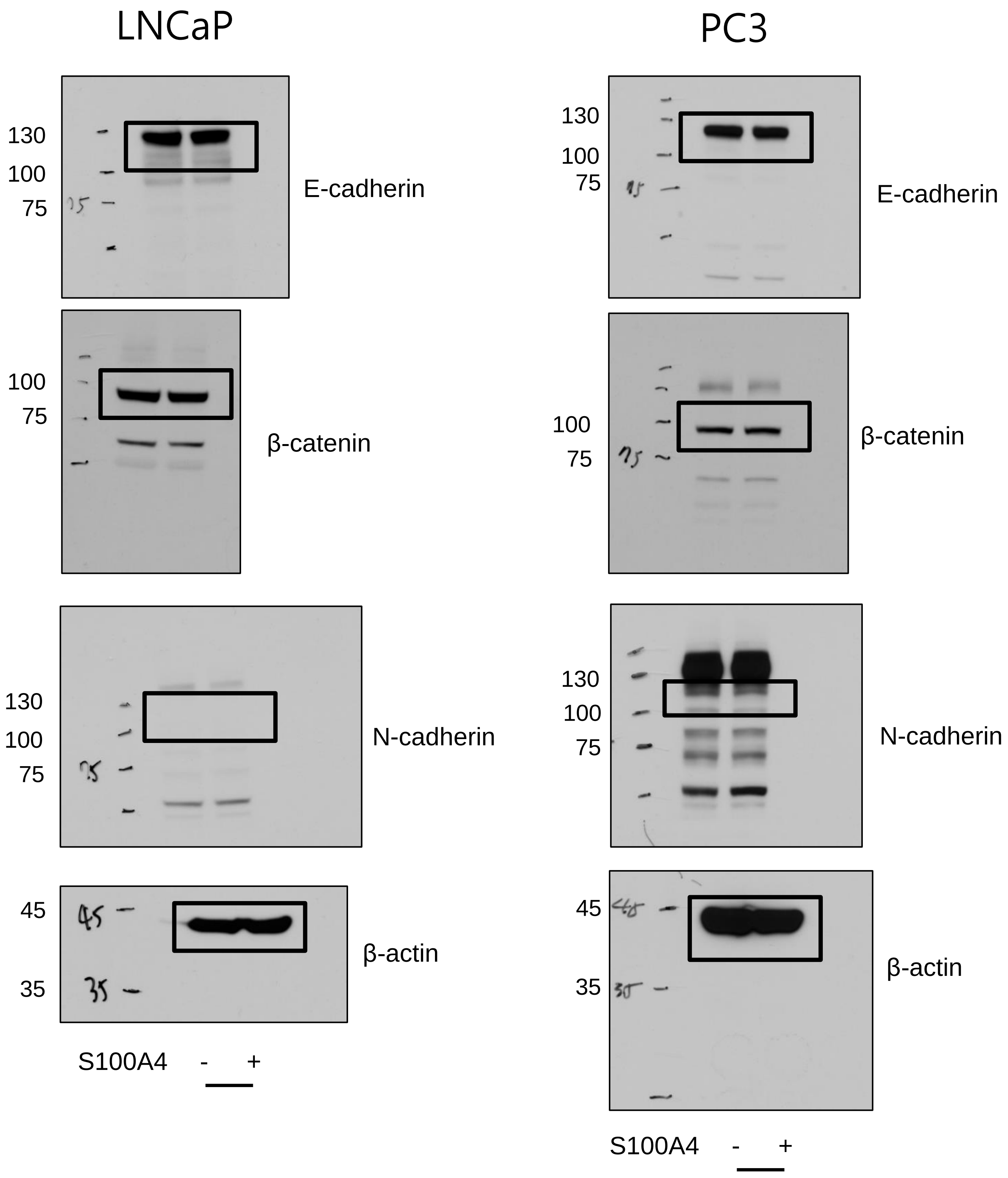

g Figure 5e uncropped images

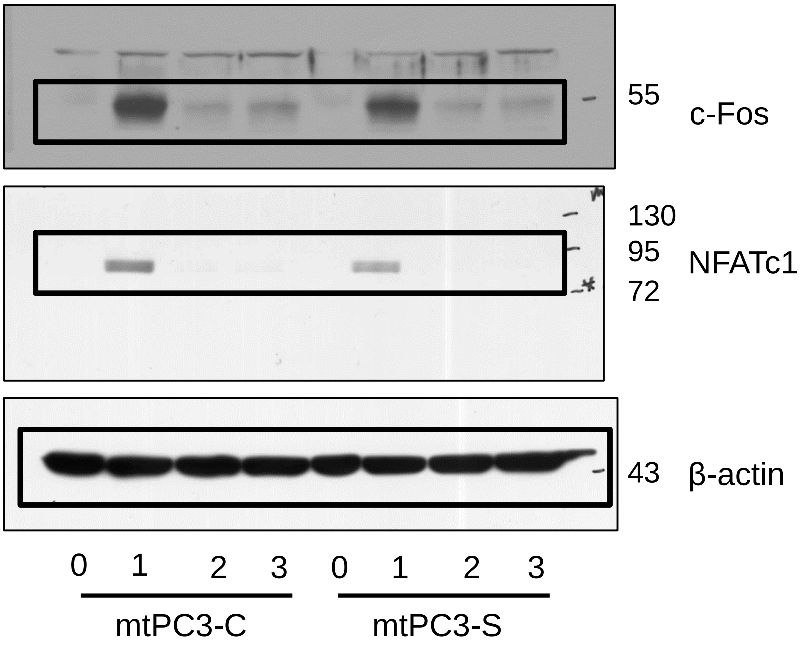

h Figure 6a uncropped images

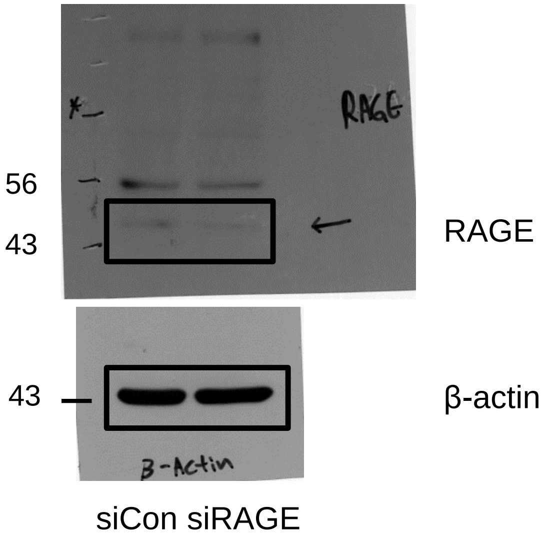

i Figure 6d uncropped images

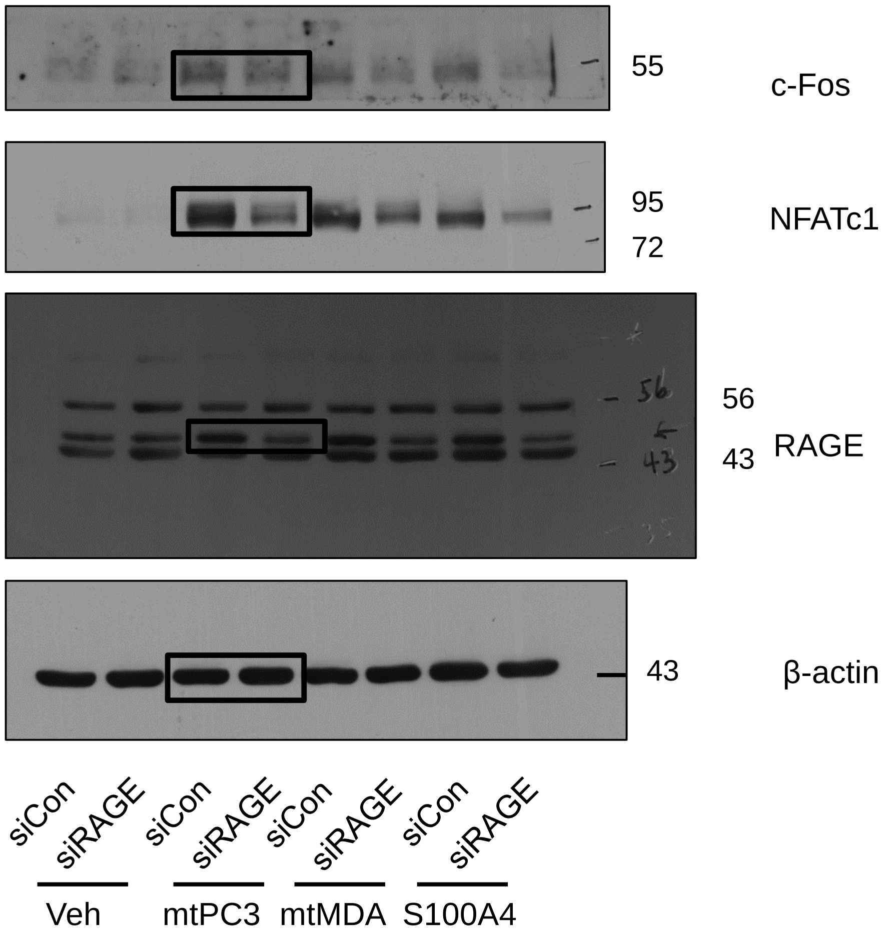

Supplement: Supplementary file 3 — Additional file 3: Supplementary Fig. 3. The uncropped full-length western blotting images of figures. [file 12885_2021_7850_MOESM3_ESM.pdf]
